# A Review on Apple Pomace Bioactives for Natural Functional Food and Cosmetic Products with Therapeutic Health-Promoting Properties

**DOI:** 10.3390/ijms251910856

**Published:** 2024-10-09

**Authors:** Maria Vandorou, Christos Plakidis, Ilektra Maria Tsompanidou, Theodora Adamantidi, Eirini A. Panagopoulou, Alexandros Tsoupras

**Affiliations:** 1 Hephaestus Laboratory, School of Chemistry, Faculty of Sciences, Democritus University of Thrace, Kavala University Campus, St. Lukas, 65404 Kavala, Greece; mavando@chem.ihu.gr (M.V.); chrplak@chem.ihu.gr (C.P.); iltsoba@chem.ihu.gr (I.M.T.); theadam@chem.ihu.gr (T.A.); 2 Department of Dietetics and Nutrition, Harokopio University, 70, El. Venizelou Ave., 17676 Kallithea, Greece; epanagop@hua.gr

**Keywords:** apple, apple pomace, phenolics, polar lipids, unsaturated fatty acids, anti-inflammatory, antioxidant, anti-aging, functional foods, cosmetics

## Abstract

Apples are consumed lavishly worldwide, while demand is increasing for the management of the huge apple-waste amounts that lead to significant disposal costs and ecological issues. Additionally, apples represent fruits with several bioactive constituents, which are key factors in a healthy, balanced diet. In the present study, an extensive review is presented regarding the bioactive compounds of an apple processing by-product, namely apple pomace, mentioning their significance as viable ingredients/substances in foods and cosmetics aiming at chronic disease prevention and health promotion. Apple pomace contains several constituents, such as polar lipids, phenolics, vitamins and dietary fibers, with potential antioxidant, anti-inflammatory, anti-thrombotic, anti-aging and skin-protecting properties, and thus, they may contribute to minimizing the risk of various health conditions. Additionally, the mechanisms of action of such functional bioactives from apple pomace exert health benefits that will be examined, while the potential synergistic effects will also be investigated. Moreover, we will present the methods and techniques needed for the utilization of apple pomace in the appropriate form, such as powder, extracts, essential oil and so on, and their several applications in the food and cosmeceutical industry sectors, which summarize that apple pomace represents an ideal alternative to synthetic bioactive compounds.

## 1. Introduction

“An apple a day keeps the doctor away”, according to the moto, which highlights the significance of apple consumption in health promotion and well-being [1]. Apples naturally stand out as one of the most widely consumed fruits worldwide, since they are highly adaptable and easily accessible [1,2]. Additionally, apples contain several essential macro- and micro-nutrients, namely carbohydrates, minerals and vitamins. The aforementioned motto points out the myriad health benefits linked to the intake of apples [1], while the same is true for fruit and vegetable consumption in general. Thus, the rising awareness of these health benefits has played a pivotal role in the increasing interest and popularity of vegetables and fruits, including apples. As a result, the cultivation of apples has spread throughout the world, and their popularity has increased rapidly, since the apple industry is estimated to be worth $10 billion globally [1,3,4].

Apples, apple products such as cider, juice, and vinegar, and apple by-products, including apple pomace, may represent essential components of health-conscious dietary patterns, focused on minimizing the risk of various diet-related conditions, namely obesity, diabetes and cardiovascular diseases, especially in the developed world, including Western and Westernized societies [4,5]. Apple pomace is the main apple processing by-product and consists of apple calyx, core, peel, seeds and stem, while it also contains the processed soft tissue, namely mesocarp; its content makes it a diverse mixture of apple constituents. Apple pomace has ≥66% moisture content, and thus it is easily perishable [6]. Even though the differentiations in several factors, such as apple variety, pedoclimatic conditions and processing methods, may affect the physicochemical composition of apple pomace, it is obvious that it is rich in several macronutrients and bioactives, namely fibers, minerals, polysaccharides, lipid bioactives, polyphenols, vitamins, triterpenes and pigments [2,4].

Currently, apples and apple products are consumed lavishly worldwide, and thus, there is a growing demand for the management of the rapidly increasing apple-waste amounts that lead to significant disposal costs and several ecological issues. Although apple pomace is often regarded as waste and disposed of, as an apple by-product, it constitutes a valuable reservoir of bioactives, including fibers, pectins, and microconstituents, such as polar phenolics and lipid bioac-tives [2,4], that are well-known for their potential health benefits, including their antioxidant activity and anti–inflammatory properties [4,7]. Consequently, food industries should apply the appropriate methods for the recovery of the aforementioned bioactives and for their utilization as functional ingredients in functional products with added value and potential health-promoting properties in the food, cosmeceutical and nutraceutical sectors [2,4].

All in all, the present review sums up the health benefits of apple pomace bioactives and their abilities to alleviate and prevent chronic inflammation-linked diseases; efficient methods for the extraction and recovery of apple pomace and its bioactives are also discussed. The utilization of apple pomace or its bioactive compounds for the fortification of functional foods and cosmetics, dedicated to combating chronic diseases, all while maintaining the sensory attributes and overall excellence of the products, will be mentioned as well in the present manuscript.

## 2. Materials and Methods

A comprehensive literature survey was performed using scientific databases such as Google Scholar, Web of Science, and Scopus. Data were gathered from scientific publications in English from 2018 to 2024 using the following keywords: apple pomace, by-products, nutritional composition, bioactive compounds, health, foods and cosmetics. The inclusion criteria were based on the contents such as (i) the design of the experimental study, (ii) health benefits, (iii) food application and (iv) cosmetics. In contrast, studies were accepted if they were in editorials, letters, short surveys, reviews, patents, and conference abstracts. Thus, articles were selected that described the nutritional composition and the chemistry of apple pomace’s bioactive compounds, health-promoting biological activities, and the impact of apple pomace’s bioactive compounds on foods and mainly on cosmetics.

## 3. Apple Pomace Composition

### 3.1. Apple Pomace Macro-Constituents

Apple and apple by-products, such as apple pomace, are sources of macro-constituents such as carbohydrates and dietary fiber [4,8]. As a result, apple pomace is a rather precious fraction of the food industry with plenty of health-promoting properties that needs to be recovered, instead of being treated as waste. Specifically, apple pomace contains simple soluble sugars, including fructose, glucose and galactose (~23%, 22% and 6–15%, respectively, or 45.1–83.8% carbohydrates in general on a dry basis) (Table 1) [9]. Additionally, apple pomace contains significant amounts of dietary fibers (up to 65% on a dry basis), among which the majority are the insoluble ones, namely cellulose, hemicellulose and lignin; cellulose is the predominant fiber (~43%), which is followed by the hemicellulose (varies among ~20–32%) (Table 1) [9,10]. Apple seeds are also present in apple pomace, and as a result it contains remarkable amounts of proteins and lipids (~49% and 24%, respectively) [10]. Additionally, apple pomace contains constituents, namely pectins and starch, which make it a potent ingredient of foods, such as jellies and jams, due to their ability to enhance thickness [4].

### 3.2. Apple Pomace Micro-Constituents

**Figure 1:**
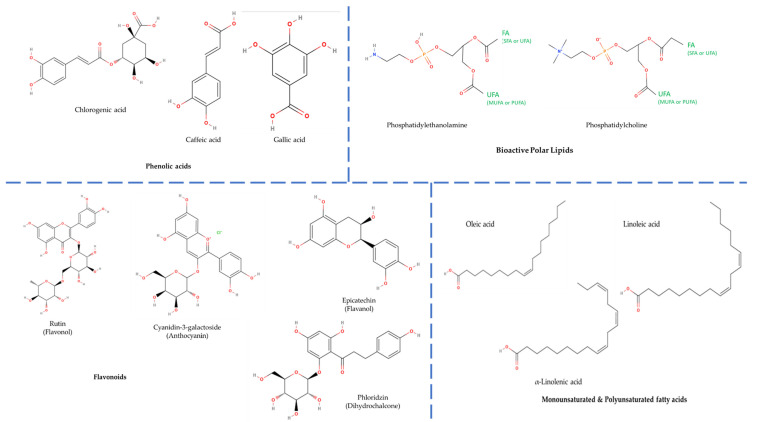
The most characteristic phenolic and lipid bioactives associated with the diverse bio-functional health-promoting properties of apple pomace. (Abbreviations: FA = fatty acid; SFA = saturated fatty acid; UFA = unsaturated fatty acid; MUFA = monounsaturated fatty acid; PUFA = polyunsaturated fatty acid). The structures for each individual bioactive were obtained from https://molview.org; accessed on 8 July 2024.

Apple pomace is a valuable source of micro-constituents such as polyphenols, carotenoids and phytosterols [4,8]; additionally, it contains several lipid bioactives, polar phenols and tocopherols [4,12,13] (Figure 1, Table 1) **[9]**. The aforementioned macro- and micro-constituents are crucial for the human metabolism function and their health-promoting properties, such as anti-cancer, anti-histaminic, anti–inflammatory, antioxidant, antithrombotic and anti-tumor properties, and hence, apple pomace is of great interest for the researchers [3,12,13]. The amounts of bioactives in apple pomace depend on several factors, including apple variety, pedoclimatic parameters and the applied production method [12]. For example, during apple cider production, producers opt to remove the seeds, skin and apple stems before the pressing process, and as a result, the apple pomace contains only few apple-derived bioactives [14].

Obviously, apple pomace is a rather beneficial and promising by-product, which is of global interest when added or supplemented in several functional products, such as nutraceuticals, pharmaceuticals, cosmeceuticals and foods [4].

## 4. Apple Pomace: Constituents with Health-Promoting Properties

### 4.1. Apple Pomace Polyphenols: Mechanisms of Action and Health Promotion

Apple pomace contains several polar phenolic classes, mainly tannins [2,4]. Tannins are compounds responsible for the astringency and bitterness in the taste of apples, and hence, they are valuable micro-constituents for the apple cider industry; on the other hand, bitterness is an undesirable characteristic for the apple juice industry. It is worth mentioning that the ripening and fermentation of apples significantly affect the tannin amounts, which accounts for the apple pomace as well [15].

Regarding the individual apple parts, peels are the main source of polar phenolics, indicating that apple pomace contains higher amounts of such compounds compared to the whole fruit itself [6]. Apple pomace polyphenols, namely flavanols, dihydrochalcones, quercetin glycosides and phloridzin, among others, are the ones possessing health benefits and antioxidant activity [3,9]. These micro-constituents are able to convert apple pomace from a by-product to a natural source of antioxidants, which are able to inhibit the reactive oxygen species (ROS), and as a result the cascade of free radical reactions is interrupted, particularly in lipid peroxidation. Such actions of the polyphenols contained in the apple pomace may lead to exceptional results when utilizing apple pomace in cosmeceuticals for dermatological uses. Additionally, the sensory characteristics of functional foods, namely aroma, color and flavor, may positively alter after apple pomace addition, while simultaneously enhancing their health-related aspects [2]. The exact quantity of apple pomace to be added in the respective functional food is of great significance, since a balance between the enhancement of health-promoting properties and the sensory characteristics should be maintained.

Except for the antioxidant activity of the polar phenolics of apple pomace, such compounds have also exhibited several health-beneficial traits such as anti-diabetic, antimicrobial, anti-inflammatory, anti-tumor and cardioprotective properties. Thus, apple pomace itself, and its polyphenolic extracts, are rather vital as functional ingredients of cosmeceuticals, pharmaceuticals and food products [16]. In the same time, there is an increasing interest regarding the utilization of phenolic compounds derived from apple pomace for the development of polyphenol-rich biomaterials suitable for several applications in the biomedical pharmaceutical sectors [17].

According to animal studies conducted on mice with cardiovascular diseases, when polyphenol-rich extracts that derive from apple peels were added in their diet, notable improvements on blood pressure, endothelial function, insulin sensitivity and lipid balance were detected in the intervention group, compared to the control one [10]. Additionally, apple pomace polar lipids and especially phenols, which are predominantly located in the peel, including chlorogenic acid, cyanidin-3-galactoside, phloridzin, rutin and epicatechin, in addition to preventing cardiovascular diseases, diabetes and cancer, has also been observed inducing asthma prevention [18]. Furthermore, it has been demonstrated that apple pomace extract, rich in dihydrochalcones, may effectively contribute to the management of several inflammation-related diseases, including type-2 diabetes, hyperglycemia, and obesity; such findings may indicate that these microconstituents can participate in sedum production regulation and thus, in the alleviation of painful skin issues, such as acne, which is characterized by irregular fat production [17]. As for the phloridzin, it has been proved that it may possess several powerful properties, such as anti-diabetic, anti-inflammatory, antioxidant, and antimicrobial, among others, and thus phloridzin might be a valuable ingredient in both the food and pharmaceutical sectors [10,13]. Alongside, simple phenolic acids like caffeic acid and gallic acid, traced in apple pomace, have been demonstrated to display antioxidant potential by scavenging free radicals via the donation of hydrogen atoms [13]. As previously mentioned, apple pomace also contains chlorogenic acid, a potent antioxidant proven to exhibit neuroprotective properties when added to foods [10]. Moreover, it has been stated to possess the capacity to mitigate hypertension by reducing blood pressure [10]. Another apple pomace bioactive is epicatechin, which possesses very strong antioxidant and anti-inflammatory attributes; at the same time, it may contribute efficiently to addressing cancer, managing diabetes, preventing cardiovascular diseases, serving as a neuroprotective agent, and improving altogether muscle performance and overall health [10].

Apples and apple-derived products are rather rich in dietary fibers, which, along with the polar phenolics, may exhibit potent prebiotic properties. In addition, apples’ soluble and insoluble fibers contribute significantly to a lower glycemic index [10]. All the aforementioned polar phenolics of apple pomace have been effectively extracted using organic solvents, such as ethanol and methanol, while several alternative environmentally friendly extraction methods, including enzymatic, microwave, and ultrasound extraction and maceration, have also been applied [2,19,20]. However, there is a need for food-grade solvents, namely food-grade ethanol and water, to be used for the extraction of such micro-constituents prior to their incorporation in foods and/or cosmetics. Recent studies investigated two novel techniques for the effective extraction of apple pomace polyphenols, namely ethanol solvent extraction and subcritical water extraction, while different apple parts and their combinations were evaluated, such as core, pulp, peel, seed, and stem. The findings underscored the richness of apple pomace as a source of polyphenolic compounds with potential applications in biomedicine, while among the several fractions collected, the subcritical water ones exhibited comparable efficacy to ascorbic acid and all ethanolic extracts, and were found to be compatible with human fibroblast (3T3-L1) and salivary gland acinar cells (NS-SV-AC) [2,19,20].

Nevertheless, more information is needed regarding the effectiveness of the aforementioned methods when applied in the apple pomace itself, while the extraction of the bioactives from apple pomace should be an eco-friendly process, achieving the dual objectives of extracting valuable compounds and reducing environmental risks [13].

Interestingly, only recently, some food grade extracted bioactives from apple pomace have shown potent anti-inflammatory and antithrombotic potency against the thrombo-inflammatory pathways of the platelet activating factor (PAF) and adenosine 5′-diphosphate (ADP) in human platelets, as well as potent antioxidant effects when assessed in vitro, with the ferric-reducing antioxidant power assay (FRAP assay) and the 2,2′-Azinobis-(3-Ethylbenzthiazolin-6-Sulfonic Acid) assay (ABTS assay), compared to a Trolox (water–soluble vitamin E analog) standard. A structural analysis of these food-grade extracts from apple pomace revealed that they contain mostly amphiphilic bioactives like phenolic and polar lipid bioactives, rich in unsaturated fatty acids like the omega-3 alpha linolenic acid (ALA; 18:n3) and the omega–9 oleic acid (18:1), as well as a considerable amount of carotenoids. The presence of all these bioactives in such food-grade extracts of apple pomace provides an explanation for their observed strong anti-inflammatory, antithrombotic and antioxidant capacity. Furthermore, the enrichment of whole grain breads with these bioactive apple pomace extracts enhanced the bio-functionality for these bakery products by increasing their anti-inflammatory, antithrombotic and antioxidant health benefits [21]. These outcomes outline that the food-grade extractions of apple pomace should not only focus on obtaining its bioactive phenolics, but also on obtaining the whole gamma of bioactives present in this sustainable natural source for developing novel natural products with health-promoting properties.

### 4.2. Additional Macro- and Micro-Constituents in Apple Pomace with the Potential to Promote Functional Health—Mechanisms of Action

Even though apples and apple-derived products (i.e., apple juice and cider), as well as by-products (i.e., apple pomace), have low lipid content, recent studies have unveiled that they contain amphiphilic compounds with remarkable bioactivities against thrombo-inflammatory mediators, namely ADP and PAF [21,22,23]. Such findings are of great importance, since some of the apple’s hydrophilic compounds had remarkably lower anti-inflammatory, antioxidant, and anti-platelet efficacy, when compared to other fruits, such as tomatoes, grapefruits, melons, and strawberries [24]. Thus, the amphiphilic compounds of apple, its juice, and cider products and apple pomace, such as phosphatidylcholines (PC) and phosphatidylethanolamines (PE), rich in unsaturated fatty acids, were the most bioactive compounds from these sources with a mechanism against human platelet aggregation, induced by these thrombo-inflammatory mediators [21,22,23].

The total lipids of apple pomace, along with polar and neutral ones, vary depending on the apple variety from which the pomace is produced. It is worth mentioning that the total lipid content, and thus the polar lipids of apple pomace, are significantly higher than those in apple cider or juice, indicating that apple pomace may be a valuable, sustainable natural source of such lipid bioactives, containing ~2–5 g of total lipids/100 g of apple pomace, 80% of which are polar [12,22]. As already mentioned in the present paragraph, the polar lipids of apple pomace were found to exhibit anti-inflammatory properties via the effective targeting of the thrombo-inflammatory mediator PAF, and hence, inflammation and its associated manifestations were enhanced [12]. Additionally, apple pomace polar lipids demonstrated significant anti-platelet effects on human platelets, mainly affecting the ADP pathways [12]. As for the polar lipid fraction of apple pomace, it was notably rich in unsaturated fatty acids, particularly omega-3 (n-3), i.e., ALA, omega-6 (n-6), i.e., linoleic acid (LA; 18:2n6), and the omega-9 oleic monounsaturated fatty acid, among others [12,21,25]. These compounds may contribute to the anti-inflammatory attributes of apple pomace polar lipids [12]. With polar lipids constituting up to 80% of the total lipids in apple pomace, the extraction of these bioactives has become more accessible, facilitating the production of novel functional products [5,10,21].

At the same time, the myriad health benefits and their key roles in cellular structure and metabolism make such compounds stand out as valuable components in the production of functional products, while they may also enhance sensory characteristics, like the nutritional value and texture [4,12]. Additionally, the strong inhibition of ADP and PAF by the bioactive polar lipids of apple pomace suggests that these constituents may be a part of novel recipes with great health-promoting properties against numerous inflammation-related disorders, such as allergies, autoimmune diseases, cardiovascular diseases, cancer, persistent infections, renal and neurodegenerative disorders [5,26,27,28,29].

Whole grain breads are some of the novel functional products, which include apple pomace extracts rich in bioactives that exhibit substantial anti-inflammatory, antioxidant and antithrombotic benefits. At the same time, apple pomace is a source of dietary fibers, due to the apple’s cell wall structure, including compounds such as cellulose, lignin and pectin [3]. Dietary fibers are macro-constituents with several health-promoting abilities, such as reducing blood cholesterol levels, ischemic heart disease and cancer’s appearance [30]. Due to its powerful structural properties, dietary fiber may be utilized to enhance sensory attributes, specifically the texture and viscosity of novel products [30]. Additionally, apple pomace dietary fibers may be exploited as carriers for several bioactives, such as polyphenols, in functional products, and thus, their potential as functional foods and cosmeceuticals holds serious promise [31]. In addition, apple pomace contains several aromatic constituents, like the benzoic acid and 2-phenylacetic acid, which may contribute to boosting sensory characteristics, such as aroma and flavor when used in foods and cosmeceuticals [32]. Among other possibilities, the jelly products industry may incorporate pectins derived from apple pomace, as this has led to both an increase in bioactives’ levels and an enhancement of the final product’s appearance [33,34].

### 4.3. Mechanisms of Action of Natural Amphiphilic Bioactives (Rich-in-UFA Biofunctional Polar Lipids and Phenolic Bioactives) from Apple Products and By-Products against Oxidative Stress and Thrombo-Inflammatory Signaling

**Figure 2:**
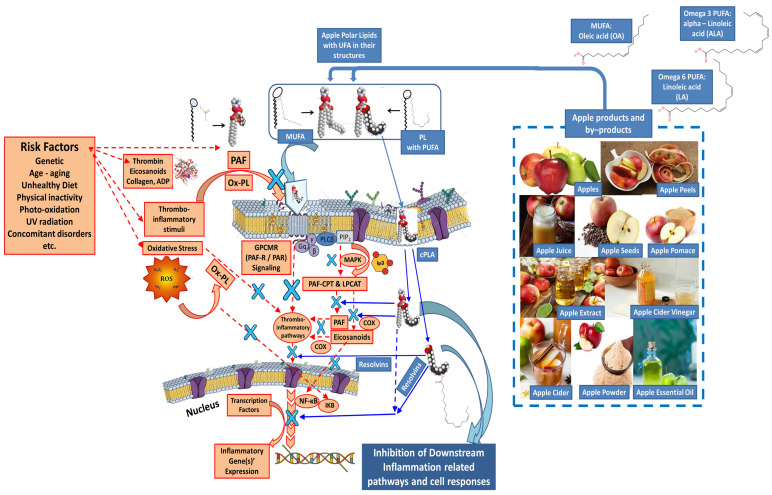
The mechanisms of action of biofunctional polar lipids with UFA in their structures, derived from apple products and by-products, against thrombo-inflammatory signaling and associated inflammatory cell responses and manifestations. Red colors: the representative signaling of thrombo-inflammatory stimuli induced by the presence of several risk factors, such as those of PAF and thrombin, which, via the pathways shown, propagate the inflammatory cell responses. Blue colors: Apple pomace bioactive polar lipids (PLs) rich in UFA beneficially affect all these signaling pathways and transcription factors, and thus further inhibit the expression of thrombo-inflammatory genes and associated cell responses (The blue X represents an inhibitory effect on a pathway and/or an enzyme and/or a receptor and/or a transcription factor and/or the expression of thrombo-inflammatory genes by apple pomace bioactive polar lipids, as indicated by the blue arrows). Abbreviations: UFA = unsaturated fatty acid; MUFA = mono-unsaturated fatty acids; PUFA = poly unsaturated fatty acids; PAF = platelet-activating factor; GPCMR = G-protein-coupled membrane receptors; PAF-R = PAF receptor; PAR = protease-activated receptors for thrombin; PAF-CPT and LPCAT = the basic biosynthetic enzymes of the two distinct pathways of PAF synthesis, PAF-cholinephsphotransferase and lyso-phosphatidylcholine acetyltransferase 2, respectively; cPLA = cytoplasmic phospholipase A2; MAPK = Mitogen-activated protein kinase; IP3 = Inositol trisphosphate; OA = oleic acid; ALA = alpha-linolenic acid; COX = cycloxigenase; NF-kB = nuclear factor kappa beta; IKB = inhibitor of NF-kB; ROS = reactive oxygen species; PL = polar lipids; Ox-PL = oxidized phospholipids; ADP = adenosine diphosphate; PLC = phospholipase C. (the cell/nucleus membranes depicted were reproduced from https://mindthegraph.com/; accessed on 25 July 2024).

**Figure 3:**
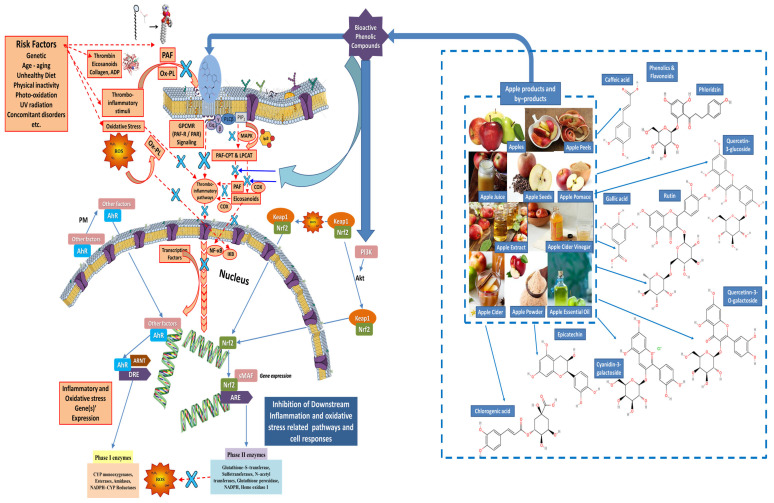
Representative mechanisms of the antioxidant and anti-inflammatory actions of phenolic bioactives from apple products and by-products against oxidative stress and thrombo-inflammatory signaling associated with aging and chronic disorders. Red colors: the signaling of oxidative stress and thrombo-inflammatory stimuli induced by aging-related risk factors, such as the induction of oxidative stress and the thrombotic and inflammatory pathways of PAF and thrombin that are associated with aging processes and chronic disorders. Blue colors: Apple pomace phenolic compounds affect all these signaling pathways related to oxidative stress, inflammation, aging and associated chronic disorders through the deactivation of ROS, the inhibition of oxidative damage to DNA, proteins and lipid molecules, and especially to the polar lipids in cellular and intracellular membranes, as well as through the inhibition of oxidized PLs, PAF- and thrombin-binding on their receptors, and thus through the reduction in their thrombo-inflammatory signaling and their aging-associated manifestations. (The blue X represents an inhibitory effect on a pathway and/or an enzyme and/or a receptor and/or a transcription factor and/or an expression of a gene related to oxidative stress and inflammation by apple pomace bioactive phenolics, as indicated by the blue arrows). Abbreviations: PAF = platelet-activating factor; GPCMR = G-protein-coupled membrane receptors; PAF-R = PAF receptor; PAR = protease-activated receptors for thrombin; PAF-CPT and LPCAT = the basic biosynthetic enzymes of the two distinct pathways of PAF synthesis, PAF-cholinephsphotransferase and lyso-phosphatidylcholine acetyltransferase 2, respectively; cPLA = cytoplasmic phospholipase A2; MAPK = mitogen-activated protein kinase; IP3 = inositol trisphosphate; OA = oleic acid; ALA = alpha-linolenic acid; COX = cycloxigenase; NF-kB = nuclear factor kappa beta; IKB = inhibitor of NF-kB; ROS = reactive oxygen species; PL = polar lipids; Ox-PL = oxidized phospholipids; ADP = adenosine diphosphate; PLC = phospholipase C. (the cell/nucleus membranes depicted were reproduced from https://mindthegraph.com/; accessed on 25 July 2024).

Chronic uncontrolled inflammation induced by the presence of several aging-related risk factors (i.e., age, unhealthy dietary habits, physical inactivity, abuse of alcohol, smoking, etc.), usually results in oxidative damage by harmful reactive oxygen and nitrogenous species (ROS and NOS) and in the continuous increase in the levels of thrombo-inflammatory mediators, chemokines and cytokines, like PAF, interleukins 1β and 6 (IL-1/IL-6), tumor necrosis factor α (TNFα), vascular endothelial growth factor (VEGF), etc., as well as the expression of associated genes for the production of aging-related inflammatory peptides and proteins, including such chemokines and cytokines and their receptors, but also enzymes that further induce aging and inflammation-related oxidative damage and extracellular matrix degradation, such as several metalloproteinases, cyclooxygenases (COX) 1 and 2, lipoxygenases (LOX), collagenases, elastases, etc. (the red-colored mechanisms in Figure 2 and Figure 3).

With respect to thrombo-inflammatory stimuli, PAF has been characterized as one of the central links of all inflammatory cell responses related to inflammation, thrombosis and aging-related manifestations associated with the induction, propagation and development of chronic disorders [35,36]. The PAF- and thrombin-signaling pathways are initiated after their binding in their G-protein-coupled membrane receptors (GPCMR), which propagate cascades of further PAF synthesis, by induction of PAF-basic biosynthetic enzymes, protease-activated receptors for thrombin (PAF-CPT) and lyso-phosphatidylcholine acetyltransferase 2 (LPCAT), as well as the production of several pro-inflammatory eicosanoids and prostaglandins by COX 1 and 2 activation, while all of these steps and these inflammatory mediators facilitate, through a vicious cycle, the propagation of the thrombo-inflammatory signaling and the expression of inflammatory genes, concluding in downstream inflammatory activation and associated cell responses. Oxidative stress also induces the aforementioned pathways, i.e., through the production of oxidized membrane polar lipids (Ox-PLs), which imitate the pathogenic role of PAF on its receptor and thus further induction of the inflammatory signaling, while the production of ROS can also facilitate the production of Ox-PL and the expression of inflammatory genes, which further propagate the inflammatory cell responses [22,24].

With respect to oxidative stress and the associated mechanisms of oxidative damage induced by the presence of risk factors related to aging and inflammation (Figure 3), they are also associated with activation of eicosanoids, and the Ox-PL formation and activation of their associated thrombo-inflammatory stimuli, as well as nuclear factor kappa B (NF-κΒ) activation, the expression of inflammatory genes, the formation of super reactive intermediates such as NOS and ROS, which are the primary external components of oxidative stress that can irreversibly damage vital biomolecules, including lipids, proteins, and DNA, and further induce the expression of oxidative enzymes and mediators of thrombo-inflammatory stimuli and extracellular matrix degradation (the red-colored mechanisms in Figure 3).

Bio-functional polar lipids (PLs) from apples, apple products and by-products like apple pomace have been found to beneficially affect the inflammatory signaling of PAF and of Ox-PLs by inhibiting the binding of PAF and/or Ox-PLs on a specific G-protein-coupled membrane receptor (PAF-receptor; PAFR) and thus, the PAF-related thrombo-inflammatory signaling that is involved in the aforementioned pathways [4,12,21,22,23]. PL bioactives from apple products and by-products beneficially affect all these signaling pathways, not only through the inhibition of PAF- and thrombin-binding on their receptors and/or through a reduction in the thrombo-inflammatory signaling (after PLs are infused within the cell due to their amphiphilic nature and after their rich UFA content is released by the action of cPLA2), but also by modulating the PAF metabolism and COX 1 and 2 activities to reduce the levels of PAF, eicosanoids and prostaglandins, and increase the levels of resolvins, and thus further inhibit the action transcription factors and the expression of thrombo-inflammatory genes and associated cell responses [22,23,24]. The representative anti-inflammatory activities of such PLs rich in UFA, derived from apple products and by-products, against the thrombo-inflammatory stimuli of PAF and/or Ox-PLs induced by the presence of aging-related risk factors, are shown in Figure 2 (the blue-colored mechanisms in Figure 2).

Apart from the PLs, several bioactive phenolics from apples, apple products and by-products have also shown strong anti-inflammatory properties against the PAF/PAFR-signaling, PAF synthesis and all PAF-related thrombo-inflammatory signaling pathways, as well as by inhibiting inflammatory responses via the MAPK signaling pathway and the inhibition of NF-κB activation and expression of inflammatory genes (Figure 3). Moreover, such phenolic bioactives can help to protect from oxidative stress as potent antioxidant compounds by inhibiting ROS production and by further suppressing the pathways involved in NF-κB activation and the expression of enzymes and mediators associated with aging, oxidative stress and inflammatory cell responses and manifestations, including COX 1 and 2, 5-LOX, peroxidases, matric metalloproteinases (MMPs), elastases, collagenases, etc.

These bioactive microconstituents act as potent radical scavengers and hydroperoxide neutralizers, while also possessing the ability to function as metal chelators, converting metal prooxidants into stable compounds. At the same time, such bioactives enhance the activity and expression of antioxidant, anti-aging, and anti-inflammatory enzymes, such as phase II detoxifying enzymes like glutathione S-transferase, NAD(P)H-quinone oxidoreductase, and UDP-gluronosyl transferase, which have antioxidative properties. They also promote the synthesis of elastin and collagen for extracellular matrix regeneration, as well as the anti-PAF enzyme LpPLA2, which degrades pro-inflammatory factors (the blue-colored mechanisms in Figure 3) [37,38,39,40].

The antioxidant activity of phenolics is largely influenced by the number and position of hydroxyl (OH) groups and by their structures in general. Flavonoids, i.e., phenolics with more hydroxyl aromatic rings, tend to show improved antioxidant properties. Thus, phenolics may be both beneficial and harmful—acting as antioxidants to combat oxidative stress, but also displaying prooxidant activity when consumed in excess—since when phenolics lose an electron, the molecule tends to become a radical. However, most studies on their prooxidant effects have been conducted in vitro, while the in vivo ones are scarce. In addition, their low bioavailability and the metabolic changes they undergo during digestion may lead to a reduction in their effectiveness in vivo, compared with the in vitro action. Thus, further research is needed to fully understand which flavonoids act as antioxidants or prooxidants in the human body [37,38,39,40].

Ingested polar phenolics and their metabolites indirectly affect aging-related oxidative stress and inflammatory signaling more effectively, since they affect the gene expression and the transcription factors at the cellular level (Figure 3). Such effects occur at much lower concentrations than the ones used in vitro against ROS. Phenolic compounds, such as those derived from apples, have a significant impact on the expression of antioxidant enzymes, which is regulated by the transcription factor Nrf2(Figure 3). Since most of Nrf2’s targets are antioxidant and cytoprotective enzymes, Nrf2 plays a key role in the regulation of the cellular antioxidant responses and it is a crucial target for the polar phenolic compounds which help in the protection against oxidative stress-related diseases [37,38,39,40].

Nrf2 translocates into the nucleus and regulates genes’ transcription encoding antioxidant enzymes through the antioxidant-responsive elements (ARE) pathway. A low level of oxidative stress is enough for the normal cell function, while excessive oxidative stress and inflammation can lead to disease. Nrf2 reduces ROS production, protecting cells from oxidative damage [37,38,39,40]. More specifically, the Nrf2-Keap1 pathway that usually regulates redox balance and detoxification may be activated from the phenolics, inducing antioxidant gene expression. Polar phenolic compounds, such as quercetin from apples, have shown effects on AhR-mediated signaling, further enhancing antioxidant and anti-inflammatory responses. By enhancing both the Nrf2 and AhR pathways, polar phenolics have the capacity to restore redox equilibrium and avoid inflammation. In general, natural phytochemicals, like phenolic bioactives that can react directly with Keap1’s cysteines, have the ability to activate the Nrf2 pathway, and as a result of this, these phytochemicals are referred to as indirect antioxidants [37,38,39,40].

Overall, apple pomace bioactive polar lipids and polar phenolics exhibit their anti-inflammatory and antioxidant health-promoting properties by affecting all these pathways of thrombo-inflammation and oxidative stress, and thus the associated inflammatory manifestations and cell responses linked to aging and chronic disorders [22,23,24].

## 5. Apple Pomace: A Functional Ingredient or Substrate

### 5.1. Foods

There is a plethora of studies dealing with apple pomace utilization as a functional ingredient or substrate in the food industry sector [4,21,41,42,43,44,45,46,47,48,49,50]. Specifically, apple pomace extracts rich in macro- and micro-constituents have extensively been utilized as functional ingredients in numerous food applications, such as wheat pasta, dairy, meat, whole grain bread, cookies and other bakery products [4,21,41,42,43,44], thus enhancing such products with health-promoting components, namely polyphenols, polar lipids abundant in unsaturated fatty acids and dietary fiber (Table 2) [4,21,41]. After dehydration and milling, apple pomace can be transformed into powder and produced on an industrial scale. This powder retains significant amounts of apple pomace bioactives, namely polyphenols and dietary fiber [42]. Apple pomace powder dietary fibers may be especially beneficial for consumers following gluten-free diets that frequently display a deficiency in dietary fiber. In vitro studies have demonstrated potential health benefits, including blood sugar levels improvement and weight reduction, while additional studies are needed to verify such findings of foods enhanced with apple pomace powder in human trials (Table 2) [42].

Several physicochemical, nutritional and sensory characteristics of wheat bread products have been improved after their enrichment with apple pomace powder [45] or apple pomace extracts [21]. However, it is worth mentioning that the incorporation of whole apple pomace or its derivatives in bakery products, such as powder, should take place carefully, since its high content in dietary fibers may significantly affect the final product’s sensory attributes and consumers’ acceptance and preference [33]. Instead, the incorporation of apple pomace extracts in bakery products seem to not affect the sensory attributes of the final product, while the anti-inflammatory, antithrombotic and antioxidant capacities are enhanced for the new products (Table 2) [21].

Additionally, the incorporation of apple pomace into dairy products, including yogurts, boosts their nutritional content by augmenting levels of dietary fiber and polyphenols, while it also affects their texture and sensory traits (Table 2) [46]. Apple pomace has been utilized for the enhancement of meat products as well [3,42]. For example, the addition of apple pomace to several meat products increased their dietary fiber and pectin content [9,47]; such fortifications aided in slowing down oxidative processes, reducing the accumulation of harmful substances, inhibiting the growth of microorganisms and preserving sensory qualities, particularly when used as coating and films [9,47].

Apple pomace retains several bioactive microconstituents, such as catechin, epicatechin, quercetin and its dimers (i.e., quercetin-3-glucoside, quercetin-3-*O*-galactoside), cyanidin-3-galactoside, gallic acid and phloridzin, among others; consequently, the incorporation of apple pomace powder into coating solutions helped to extend the shelf life of raw beef patties, while at the same time lower levels of lipid oxidation, a decrease in total microbial growth and a significant inhibition of the growth of *Salmonella enterica* throughout the storage period were observed in samples coated with this natural extract, compared to the control samples [48].

Apple pomace extracts or essential oils have been studied in terms of antimicrobial, anti-inflammatory, antithrombotic and antioxidant activities in vitro, while apple pomace is also used in alcoholic beverages [4,12,21,22,23]. It is worth mentioning that currently, for increasing the total phenolic content of the apple cider, there is a trend of re-using apple pomace during the fermentation process. Such a novel approach for cider production resulted not only in the enhancement of the cider’s sensory characteristics, but also in a lighter color of the final product. This process is rather valuable, since the apple pomace is a ready-to-use by-product, available immediately after cider production, with several beneficial results like waste reduction, lower disposal expenses and no further need for additional purchases and transport. Additionally, cider produced with the addition of apple pomace demonstrated an increase in their phenolic content and antioxidant capacity [51]; such findings were confirmed when wines were fortified with apple pomace in order to enhance final products’ nutritional value [33]. Therefore, this offers a promising way to improve the sensory and functional characteristics of ciders and wines, while maximizing the value of apple pomace [52].

Regarding the utilization of natural bioactives from apple products and by-products for the development of functional foods, supplements and nutraceuticals with antioxidant and anti-inflammatory health-promoting effects, an extensive discussion can be found in our previously reported book chapter [4], while in the present review the authors highlight the utilization of such compounds in the cosmeceutical sector.

### 5.2. Cosmeceuticals

As is mentioned above, there are studies dealing with apple pomace utilization as a functional ingredient or substrate in foods [4,41,42,43,44], while currently there is a rather novel approach regarding the utilization of apple pomace in the cosmeceutical industry sector. Thus, after a careful review of the recent scientific literature, a comprehensive discussion will take place on the various uses and beneficial effects of apple pomace in the production of cosmetics.

#### 5.2.1. Anti-Aging and Anti-Wrinkle Properties

Resent scientific research revealed the power of Annurca apple’s ursolic acid (UA) in cosmetic formulations. With an impressive half–maximal inhibitory concentration (IC_50_) of 286.42 mg/mL against elastase, and also enhanced by polyphenols such as quercetin, UA promised potent anti-aging effects. This bioactive compound was revealed to be safe for topical use, as it reduced wrinkles and improved skin hydration, which marked it as a valuable cosmeceutical ingredient for the achievement of youthful skin [53]. Phenolic compounds from Arisoo apples exhibited potent anti-wrinkle properties, surpassing those of Fuji apples. Ethanol extraction also yielded higher phenolic content and stronger bioactivity, compared to water extraction. Those findings suggested that Arisoo apple extracts are promising functional additives for cosmetic products targeting anti-aging management [54]. Additionally, water and ethanol extracts of ‘Summer King’ apple peel showed a notable inhibition of elastase and collagenase, indicating promising anti-wrinkle effects. The water extract demonstrated 14.55% and 67.73% inhibition, while the ethanol extract exhibited 56.50% and 77.81% inhibition, respectively. These results suggested the potential of ‘Summer King’ apple extracts for developing effective anti-aging cosmetic formulations [55]. Another study revealed that apple-derived nanovesicles (ADNVs), provided significant anti-wrinkle agents by downregulating the nuclear factor kappa B (NF-κB) pro-inflammatory pathway and promoting collagen synthesis in dermal fibroblasts. ADNVs demonstrated high effectiveness in reducing extracellular matrix (ECM) degradation and altering gene expression related to skin aging. Topical application using hyaluronic-acid-based hydrogels and patches displayed great promise for incorporating ADNVs into anti-wrinkle skincare formulations, as reported by a corresponding clinical trial [56]. Moreover, a study investigated a cosmetic cream comprising a wild apple fruit water extract, alpha-hydroxyacids (AHAs), and polyphenolic compounds for its anti-wrinkle effects, through both in vivo and in vitro assessments. An in vitro analysis revealed stable physico-chemical characteristics and high antioxidant activity during storage. In vivo tests, on the other hand, demonstrated the cream’s ability to hydrate the skin, to reduce the melanin index, and not exhibit skin irritation, highlighting its potential as an effective anti-wrinkle product with skin-beneficial properties [57]. In addition, the anti-wrinkle effects of topical formulations containing *Malus* sp. extract and rutin have been studied via in vivo and in vitro examinations. In vitro analysis results confirmed the stable physico-chemical properties of the formulations, while in vivo testing on human skin biopsies revealed an important reduction in apoptotic cells, caspase-3-positive cells, and cell population data (CPD) formation, induced by UVB irradiation. These findings underscored the potential of *Malus* sp. extract and rutin as effective agents in combating skin aging and UV-induced damage, with promising results across both experimental models [58].

Meanwhile, other studies have investigated the action of apple-derived nanovesicles (ADNVs) against skin aging. It has been found that ADNVs downregulate the action of Toll-like receptor 4 (TLR4), leading to a negative impact on the NF- κΒ pro-inflammatory metabolic pathway. ADNVs positively influenced the generation of collagen compounds (COL3A1, COL1A2, COL8A1, COL6A1) and reduced the synthesis of metalloproteinases (MMP1, MMP8, MMP9), two actions that decreased the ECM degradation on the skin. ADNVs also affected fibroblasts so as to increase the production of cholesterol. ADNVs can be formed into two types of products, gels and patches, with potential applications in both cosmetic and medicine–related industries [56].

In another study, the characterization of a cosmetic cream containing 6% wild apple fruit water extract with 3.5% AHAs and polyphenolic compounds, that was stabilized by biodegradable alkyl-polyglucoside emulsifiers both in vitro and in vivo, was conducted. The produced cream was characterized in vitro via various methods such as physicochemical analysis (pH values and electrical conductivity), antioxidant activity estimation (AA) using the DPPH test, and a determination of the amount of cosmetic active substances, including AHAs, through high–performance liquid chromatography (HPLC) analysis. Using biophysical methods, the in vivo estimation of skin irritation potential following cream application under occlusion for a 24 h period was examined on 12 healthy volunteers. The obtained results revealed no signs of skin irritation after the administration of the cream under occlusion, and that the skin’s hydration levels had increased, while hyperpigmentation was reduced [57]. The indicative anti-aging and anti-wrinkle properties of apple pomace bioactives are depicted in Table 3.

#### 5.2.2. Skin Protection

According to a recent study, the inhibition of porcine elastase enzyme activity by the Annurca apple oleolite (AAO) is primarily due to ursolic acid (UA), a potent triterpenic compound. AAO effectively inhibits elastase with an IC_50_ of 286.42 mg/mL. This inhibition is attributed to the presence of hydroxyl groups in UA and other polyphenols present in AAO, such as phloridzin, phloretin, rutin, and quercetin-3-O-glucoside. In clinical trials, AAO-based formulations demonstrated a significant reduction in nasolabial fold and forehead wrinkles, an improvement in visco-elastic parameters, and an enhancement of skin hydration, suggesting its potential as a valuable ingredient in skin protective cosmeceutical products [53,63].

Another study examined Aomori Hiba and apple polyphenol extracts for their potential as eco-friendly cosmetic ingredients. By assessing their properties and functionality, including particle size analysis and decomposition under light, the research highlighted their suitability for skincare formulations. In addition, heating apple lee powder increased polyphenol content, suggesting a pathway for enhancing skin protection through these natural extracts [59,64]. Cosmetic emulsions containing hydroalcoholic apple extract are rich in polyphenols and have displayed potential antioxidant activity for skin protection. The highest sun protection factor (SPF) (1.01) was achieved with refluxing, indicating the efficacy of apple extract in sunscreen formulations, with stability observed even after UV exposure [61]. Another research study examined the protective potential of apple extracts against UV-induced DNA damage in human fibroblasts and their potential as skin protectants. Apple extracts, particularly UFP, PF-III, and PF-IV, demonstrated significant protection against DNA damage caused by UV radiation. These extracts also exhibited strong antiproliferative effects in melanoma cells, indicating their potential use in pharmaceutical and cosmeceutical formulations for sun and skin cancer protection [65]. Moreover, UA extraction from Annurca apple using sunflower oil resulted in a potent extract named OAAO. OAAO demonstrated significant antioxidant activity and effective skin protection without adverse reactions. UA showed a linear distribution over time, indicating gradual movement from the epidermis to the dermis without entering the bloodstream, aligning with cosmetic product regulations [63].

A cosmetic cream containing 6% wild apple fruit water extract, stabilized by biodegradable emulsifiers, underwent both in vitro and in vivo characterization. In vitro analysis demonstrated stable physicochemical properties and antioxidant activity over 180 days, while the in vivo assessment revealed no skin irritation, increased hydration, and decreased melanin index after cream application. These findings suggest the cream’s potential for preventing oxidative skin damage, moisturizing dry skin, and reducing hyperpigmentation, making it suitable for cosmetic use [57]. Apple stem cells (ASCs) extract reduced inflammation, regulated collagen production, and improved skin biometrics, including thickness reduction and moisture content enhancement. Moreover, ASC application downregulated tumor necrosis factor alpha (TNF-α) expression and reduced inflammatory cell infiltration, suggesting its potential as a therapeutic agent for UVB-induced skin damage. ASC extracts are promising for clinical application in protecting the skin from UVB-induced damage [66]. Another study showed that UAE has the ability to mitigate wrinkle formation. The extract’s components, including phloridzin, interact with biological processes in the skin, such as collagen synthesis, hydration regulation, inflammation modulation, and antioxidant defense mechanisms. These interactions lead to the observed protective effects against UVB-induced skin damage, including wrinkle formation, collagen degradation, inflammation, and oxidative stress [67].

As already mentioned above, multiple studies have explored the potential utilization of apple and its by-products in cosmetics for UV protection. Apple polyphenols have shown promising properties in this regard, particularly when extracted using specific methods that yield higher SPF values. These findings highlighted the potential of apple polyphenols as natural ingredients for sunscreen formulations. Cosmetic creams containing apple polyphenols may help mitigate the harmful effects of UV radiation by blocking its penetration, thus reducing the risks associated with sun exposure. The antioxidant activity of polyphenols further enhances their efficacy in topical formulations, particularly in safeguarding keratinocyte cells against UVB-induced skin damage. This protective mechanism is attributed to both direct effects on t-BHP toxicity and indirect enhancement of antioxidant defense mechanisms within cells [61].

Considering the mechanisms in ex vivo experiments, formulations containing apple polyphenols exhibited protective effects against UVB-induced sunburn cell formation, caspase-3 activation, and cyclobutane pyrimidine dimer formation in the skin models tested. Additionally, the formulations demonstrated the inhibition of lipid peroxidation and the formation of metalloproteinases triggered by UVB radiation [58]. In addition, in vivo experiments showed that the application of ASC extract demonstrated promising results in mitigating the UVB-induced infiltration of inflammatory cells and improving collagen regulation in photodamaged skin. Notably, compared to control groups, rats treated with ASC extract exhibited a reduced thickening of both epidermal and dermal layers, indicating significant improvement in skin biometrics. Moreover, the application of ASC extract led to enhanced moisture content in the skin, which proved advantageous in treating damage and inflammation. Additionally, the expression of TNF-α, a key inflammatory marker, was notably reduced following ASC application, indicating its potential anti-inflammatory effects in treating UVB-induced damages [66].

Apart from that, other findings indicate that Unripe Apple Extract (UAE) reduced the UVB-induced secretion of interleukin 1β (IL-1β), while increasing interleukin 10 (IL-10) release. UV radiation can trigger inflammatory responses mediated by ROS through the activation of various kinase cascades, such as Akt, JNK, ERK, and p38 MAPK. Specifically, UVB irradiation activates Akt and p38 MAPK, leading to inflammatory responses and the apoptosis of keratinocytes, with p38 MAPK playing a predominant role. Thus, modulating Akt and p38 MAPK activity may help prevent UVB-induced skin damage, including oxidative stress, inflammation, and apoptosis. Furthermore, the UV-induced activation of activator protein-1 (AP-1) by MAPK promotes inflammation and collagen degradation through matrix metalloproteinase (MMP) activation. Notably, UAE downregulated AKT and p38 MAPK gene expression while upregulating tumor growth factor beta (TGF-β) gene expression, suggesting a mechanism for its protective effects against UVB-induced inflammation, collagen degradation, and apoptosis. Moreover, UAE inhibited UVB-induced skin edema and neutrophil inflammatory response by downregulating p38 MAPK gene expression. It also prevented UVB-induced wrinkle formation and skin water loss, potentially by regulating genes involved in collagen synthesis, hyaluronic acid production, and MMP activity. Additionally, UAE reduced UVB-induced skin edema, neutrophil infiltration, and IL-1β secretion while increasing IL-10 levels. It also exhibited anti-oxidative stress properties by enhancing glutathione content and inhibiting lipid peroxidation and superoxide anion production [67]. The indicative skin-protecting properties of apple pomace bioactives are presented in Table 4.

#### 5.2.3. Skin Whitening

In this context, a few components of the Formosan apple were isolated and identified. Using quantitative real-time PCR, molecular docking models, and Western blot investigations of tyrosinase-related proteins, their impact on melanin formation in human epidermal melanocyte (HEM) cells was assessed. This study has demonstrated that the antioxidant activity of apple leaf extract is extremely strong and nearly identical to that of apple fruit extract. The two most active ingredients found in the Formosan apple were 3-hydroxyphloretin and catechol, which demonstrated strong hydroxyl radical-scavenging and HEM cellular tyrosinase-reducing activity, indicating potential applications in cosmetic products as skin whitening agents [73].

Furthermore, the purpose of an interesting clinical trial was to assess the effects of continuous apple polyphenol supplementation on UV-induced pigmentation and face skin conditions in healthy women. In vitro studies revealed that apple procyanidins prevented melanocytes from synthesizing melanin and shielded against the oxidative damage brought on by UV exposure. Furthermore, they improved the intestinal gut barrier and reduced hepatic inflammation brought on by Gram-negative bacteria’s endotoxic lipopolysaccharides, both of which have anti-obesity properties. Research has indicated that alterations in the gut microbiota can impact the skin’s homeostasis mechanisms, hence mitigating the effects of ultraviolet radiation. Therefore, AP treatment may affect how the gut microbiome suppresses pigmentation and oxidation. Overall, the research points to apple procyanidins as a potential defense against UV-induced skin damage, especially in terms of keeping skin lightness and lowering pigmentation [74].

#### 5.2.4. Antioxidant Properties

Numerous studies have investigated the potential of apples and their by-products as sources of antioxidants for cosmetic applications. These studies have consistently shown that apple polyphenols possess robust antioxidant properties, which enable them to effectively neutralize free radicals and shield the skin from oxidative stress. Their compatibility with cosmetic formulations further enhanced their appeal as ingredients for sunscreens and other skincare products. The antioxidant activity stems from a variety of polyphenols present in apples, such as phenolic acids, flavonoids, and tannins. Among these, quercetin and its glycosides (isoquercetin, rutin), are particularly prevalent. These polyphenols, along with several other phenolic compounds, exhibit notable free radical scavenging activity. It is worth noting that while polyphenol content contributes to antioxidant activity, the correlation between antioxidant activity and sun protection factor (SPF) is not direct, since other compounds extracted alongside polyphenols, also influence these properties [61].

Gels and emulgels containing a complex of phenolic compounds from apple extracts have undergone an in vitro evaluation of their antioxidant activity, which was carried out via free radical scavenging activity and metal ion reducing power. Gels and emulgels enriched with apple extracts exhibited robust antioxidant properties, making them an appealing option for the development of cosmetics [62,75]. As already mentioned above, the antioxidative capacity of compounds can be demonstrated through a range of mechanisms, encompassing the inhibition of oxidative enzymes, the donation of hydrogen ions to free radicals, the scavenging of free radicals, the prevention of lipid peroxidation, and the chelation of metal ions. Antioxidants sourced from wild apple fruits exhibited interactions with free radicals, such as 2,2-diphenyl-1-picrylhydrazyl free radical (DPPH•), leading to the interruption of oxidative chain reactions. This interruption was facilitated by the transfer of hydrogen atoms from polyphenolic hydroxyl groups [68].

According to a recent study, UA was isolated from Annurca apple and turned into an extract named Optimized Annurca Apple Oleolyte (OAAO). The extract’s phenolic content and antioxidant properties were investigated. Its antioxidant activity was assessed in vitro by DPPH•, ABTS and FRAP analyses, and it was found that non-polar polyphenols and dihydrochalcones are responsible for antiradical properties. It was also found that UA is able to penetrate the skin via the intercellular route and disperse across different skin compartments [63]. In this particular study, a water/oil emulsion was synthesized, using the extract of *Malus domestica* apple. Many solvents were tested in different ratios in order to discover the best solvent for the recovery of flavonoids (antioxidant substances) from the fruit. It was revealed that methanol–formic acid–water (70: 2: 28) was the best combination, with a recovery rate of 82%. Emulsion formulas have a potential use in cosmetic and pharmaceutical industries, due to the protection they provide to encapsulated medicine. The absorption and penetration of medicaments are more easily controlled when incorporated into an emulsion. The capacity of the components, in terms of their dissemination and therapeutic qualities, were both enhanced. The action of the emulsion was prolonged and its emollient effect was greater compared to other preparations [76].

Meanwhile, in another study, the antioxidant activity of chlorogenic acid, and phloridzin and extracts themselves derived from apple’s leaves and fruit, was evaluated. More specifically, the degree of lipid oxidation was estimated through spectrophotometric and fluorimetric methods. The assays used in order to induce oxidation were UVC radiation and 2,2′-Azobis(2-amidinopropane) dihydrochloride radical (AAPH). All results were analyzed and ranked based on the effectiveness of antioxidant activity, from strongest to weakest, as follows: chlorogenic acid > apple leaf extract ≈ apple fruit extract > phloridzin. These findings indicated that apple leaf extracts possess antioxidant properties almost equal to those of apple fruit extract, and can be used for the protection of living organisms from exogenous oxidation as an ingredient in many products in the cosmeceutical or pharmaceutical industries [77].

The bioactive substances from the pomaces of four different apple varieties (Gala v. Royal Gala Tenroy, Golden v. Golden, Granny Smith, and Pink Lady v. Cripps Pink), were extracted using a variety of solvents (MeOH/H_2_O mixture, ethanol, ethyl acetate) with the microwave method. The HPLC analysis conducted on the obtained extracts, showed the existence of phenolics such as gallic acid, chlorogenic acid, catechin, rutin, phloridzin and terpenic compounds, like UA and triterpenic derivatives, with coumaryl groups. The extracts had the ability to inhibit the DPPH free radical, revealing a strong antioxidant activity [78]. This study investigated the effectiveness of two formulations with topical application consisting of 1.25% *Malus* sp. apple extract and 0.75% rutin. Hematoxylin–eosin staining of the histological sections revealed the development of cells exhibiting apoptotic characteristics after irradiation. While treatment with both formulations reduced the amount of visible apoptotic cells to a basal level, with no variation across the formulations or skin models; the radiation of both skin models caused a notable emergence of those apoptotic cells within the epidermis. When activated caspase-3 positive cells were detected by immunofluorescence, the amount of this apoptotic marker increased significantly in skin that had received radiation. For the tissue-engineered skin and cell explants, the irradiation produced 14.18% and 11.22% of caspase-3-positive cells, respectively. It is worth mentioning that the irradiated skin samples treated with both formulations did not show positive cells for caspase-3, similar to the non-irradiated skin. This suggests that rutin and the *Malus* sp. extract prevented the activation of effector caspase-3 caused by irradiation. This impact might be connected to rutin’s antioxidant potential and the *Malus* sp. extract’s ability to scavenge ROS.

Following UVB irradiation, there was a significant rise in the number of CPD-positive cells in the dermis and epidermis. Using extracts of *Malus* sp. and rutin, the number of CDP-positive cells decreased. Rutin was more effective at preventing UVB-induced CPD production than the *Malus* sp. extract. In vivo keratinocyte apoptosis is largely caused by UVB-induced DNA damage, such as CPDs. Using a skin explant model, the possible protection of rutin and *Malus* sp. extract against UVB-induced lipid peroxidation and MMP formation, two parameters associated with skin aging, was further assessed. It has been demonstrated that UVA radiation induces lipid peroxidation more effectively, by involving either a singlet oxygen or hydroxyl radical in cellular membranes. Lipid peroxidation was dramatically elevated by UVB-irradiation, rising 3.5 times faster than in the non-irradiated control group. Lipid peroxidation increased by 2.8 times in non-irradiated skin samples treated with placebo (NIP) in comparison to the cells’ basal value (NIC). It has been recently proposed that, following UVB irradiation, methyl paraben may cause lipid peroxidation in human keratinocytes. Methyl paraben was discovered in the preservative mixture of the formulations that were created. It is likely that the preservative and/or the mixture of chemicals in the formulation caused lipid peroxidation, even if no other ingredients in the formulation are known to have a pro-oxidative effect. *Malus* sp. or rutin, when added to the formulation reduced skin lipid peroxidation, confirming the effect of these compounds on the skin’s state of oxidative stress. Thus, rutin and *Malus* sp. extracts can both be useful ingredients in sunscreens that serve as photochemopreventives [58]. The indicative antioxidant activities of apple pomace bioactives are presented in Table 5.

#### 5.2.5. Antimicrobial Properties

Alongside with the antioxidant activity, the antimicrobial properties of the polyphenols were detected in the apple pomace from Belgium and Spain, and thus it can be included in many dermo-cosmetic products [82]. It has been found that many bioactive compounds abundant in apple fruit and pomace, such as phloridzin, have strong antimicrobial activity. According to a rather recent study, pure phloridzin is usually unwanted in dermo-cosmetic products, due to its quick browning, while a product derived from phloridzin called F2 has been synthesized, in which the modification has resulted in an active but also stable product, ideal for application in cosmetic formulations such as creams, lotions, masks or serums [83].

#### 5.2.6. Anti-Inflammatory Properties

After the extraction of the phenolic compounds found in apple peels of ‘Summer King’ apples, it was found that they possess anti-inflammatory, antioxidant, anti-aging and anti-diabetic properties, among others. Through the inhibition of many substances like DPPH•, FRAP, antioxidant protection factor (PF), thiobarbituric acid reactive substances (TBARs), hyaluronidase (Haase), elastase, collagenase, α-amylase α-glucosidase, apple pomace extract may be utilized and incorporated in pharmaceuticals and cosmetics with health-promoting effects [55].

Additionally, in another study, ASCs were extracted and manufactured into a product for topical application on rat skin, in order to evaluate its activity against inflammatory cells induced by UVB irradiation. The ASC extract was found to reduce UVB-induced inflammatory cell infiltration and to improve the skin’s ability to regulate collagen in photodamaged areas. An increase in the skin’s hydration level had positive clinical effects in the treatment of inflammation and injury. Furthermore, significant improvements in skin biometrics were observed, including a decrease in thicker dermal and epidermal layers, in comparison to other rat groups. The findings showed a decrease in inflammatory cell infiltration and polymorphonuclear leukocytes during the course of the seven-day treatment period. Moreover, after applying ASC, the expression of TNF-α was downregulated. In animal studies, ASC extracts may have anti-inflammatory properties and are able to treat UVB damage. These results suggested that ASC extract belongs in the category of clinical study subjects and is used in UVB-induced damage therapy [66].

### 5.3. Limitations of Apple Pomace Utilization

Even though there are several advantages considering the utilization of apple pomace in foods and cosmetics, some issues have been raised about its potential differentiations in the textural and sensory aspects of the final products, mainly regarding the food industry sector [3,53]. For this purpose, novel methods and techniques have been utilized so as to reduce such side effects, while the use of the apple pomace extracts which are rich in bioactive constituents, instead of the apple pomace itself, may be the main solution that will help reduce these negative effects without sacrificing the improved biological activities of the final products.

On the other hand, safety issues may arise regarding apple pomace consumption, since it has about 4–5% apple seeds and contains the toxin cyanogenic glycoside amygdalin, which, during digestion, interacts with several enzymes, and hydrogen cyanide is released as well, which may cause complications ranging from mild vertigo to coma and paralysis. However, it is worth noting that a very high quantity of seeds must be consumed in order to cause the aforementioned symptoms (83–500 apple seeds or >800 g of apple pomace). Additionally, methods for the removal of the apple seeds from the apple pomace have also been applied, so as to minimize their potential risk. Additionally, according to the literature, essential oil from the apple seeds is rather safe, but only within the acceptable levels of toxicity [10].

Except for some minor concerns, mainly regarding the consumption of the apple pomace, it is a suitable and valuable ingredient or substrate for the production of foods and cosmetics, while it is worth mentioning that the use of apple pomace as a functional food ingredient has been assessed as safe in accordance with environmental protection agency (EPA) guidelines on food toxins and pesticides [84].

## 6. Conclusions

Currently, the utilization of the by-product apple pomace is increasingly gaining the scientific community’s interest, as it has emerged as a valuable novel ingredient with several applications in both food and cosmetic or pharmaceutical industries. However, its use in the cosmeceutical sector is still at a very early stage, and thus more studies in this field are needed prior to mass commercialization and consumption.

Apple pomace is found to retain significant amounts of bioactive constituents, such as polyphenols and carotenoids, with potential health-promoting and chronic-disease-preventing properties, while when bioactive rich apple pomace extracts or essential oils are used instead of the apple pomace itself, the potential undesirable characteristics or projected side effects are eliminated.

Additionally, the use of apple pomace, among other by-products, except for its contribution in producing novel products with functional properties, significantly eliminates the waste by-product amounts produced by the food industry, while the disposal and transport costs are also minimized, and thus, apple pomace commercialization provides important economic benefits. For these purposes, the main strategy emphasizes driving food industries to adopt more sustainable practices by producing functional foods, cosmetics and nutraceuticals that consolidate the bioactive constituents of apple pomace. In general, such a goal highlights how significant it is to use the ready-to-use available resources, such as by-products, and given the increasing amounts of apple products, such as apple cider, apple juice, and vinegar worldwide, apple pomace’s significance is rather considerable.

According to the studies summarized in the present review, using apple pomace as a functional ingredient or substrate is a rather viable approach which represents an ideal alternative to synthetic nutrients, not only able to improve human health, but also promoting well-being, both inward via food and outward via cosmetics.

## Figures and Tables

**Table 1:** Apple pomace content and its major bioactives [4,6,9,11].

	Concentration (%) ^1^	Major Compounds
Macro-constituents		
Ash	1.5–2.5	
Carbohydrates	45.1–83.8	Galactose, Glucose, Fructose
Dietary Fibers	14.5–65.0	Cellulose, hemicellulose, lignin
Lipids	0.6–4.2	
Moisture	4.4–10.5	
Protein	1.2–4.7	
Micro-constituents		
Anthocyanins	0.005–0.013	Cyanidin-3-*O*-galactoside
Dihydrochalcones	0.069–0.254	Phloretein, Phlorizin
Flavonoids	0.215–0.373	Epicatechin, glycoconjugates, guercetin, isorhamnetin, kaempferol, procyanidin B2, rhamnetin
Phenolic Acids	0.052–0.154	Caffeic acid, chlorogenic acid, *p*-coumaric acid, *p*-coumaroyl-quinic acid, ferulic acid, sinapic acid

^1^ Dry weight basis.

**Table 2:** Applications of apple pomace or recovered functional compounds as ingredients in foods.

Foods Functional Compounds	Aims	Results	References
Wheat Pasta			
Apple pomace	Enrichment	Enrichment with phenolic acids, quercetin derivatives, flavon-3-ols, dihydrochalcones, dietary fiber	[41]
Cookies			
Apple pomace powder	50%	Increase in phenolic content and enhance of the nutritional profile	[42,43]
Gluten-free Cookies			
Apple pomace	Enrichment	Increase in levels of phenolic acids, quercetin derivatives, flavan-3-ols, dihydrochalcones	[44]
Wheat Bread			
Apple pomace powder	Enrichment	Improvements in various physico-chemical, nutritional, antioxidant, and sensory properties	[45]
Yogurts			
Apple pomace	Enrichment	- Enhancement of the nutritional value by increasing dietary fiber and total phenolic content - Affects sensory and textural characteristics	[49]
Cheese			
Apple pomace	Enrichment	- Affects composition, texture, color, overall sensory appearance - Enhances the growth of *Lactococcus lactis* strains, indicating a beneficial impact	[50]

**Table 3:** Apple pomace anti-aging properties.

Hypothesis—Intervention	Study Design/Parameters Examined	Main Findings	Year of Study	Reference
In vitro and/or ex vitro studies				
This research was dedicated to the examination of apple extracts and apple polyphenols	The minimum effective inhibition concentrations (MICs) of Hiba oil and hinokitiol were measured. Dried apple lee powder was heated (at 150, 160, 170, 180, 190, 200, 250, 260, 270, 280, 290, 300, 310, 320, 330, 340, and 350 °C) and the polyphenol quantity in the samples was measured according to the Folin–Denis method	A total of 0.38 g of polyphenol was found in the apple lees. The apple lees were also rich in sugar, and expected to maintain humidity in skin when used as raw materials for cosmetics. It was determined that sugar changed into polyphenols. Polyphenols decreased gradually after the apple lees were heated to 260 °C. This is considered to be a result of gradual carbonization of polyphenols.	2006	[59]
The effect of different drying treatments and sieving on Royal Gala apple pomace was investigated	Total phenol content (TPC) was determined, while antioxidant activity was also assessed. HPLC analysis was conducted to quantify phenolic compounds. The extraction of phenols, TPC, in vitro antioxidant activity, DPPH, ABTS and FRAP assay, as well as HPLC-UV analysis, were performed. The dimensional analysis of fractions <250 µm was performed and morphological analysis was conducted. Powder flow properties were evaluated, according to European Pharmacopoeia guidelines. Hydrogels were prepared by dispersing apple pomace in a vinegar/water mixture and NaCl solution, and their viscosity was measured.	The homogenization and oven drying at 55 °C for 12 h (HOD) sample displayed the most favorable flow properties, while the oven drying at 55 °C for 12 h (OD) sample had poor flow properties. By employing ultrasonic-assisted extraction (UAE) it facilitated the isolation of phenolic compounds, revealing differences in yield, TPC, and antioxidant assays (DPPH, ABTS, FRAP). HPLC–ultraviolet (HPLC-UV) analysis provided insight into the presence of chlorogenic acid, phloridzin, and quercetin derivatives in AP extracts. The preparation of hydrogels using the most promising samples (OD and homogenization and freeze–drying sample (HFD)) showcased their robust thickening capacity, with OD exhibiting superior performance.	2023	[60]
The purpose of this study was to evaluate the sun protection factor (SPF) of cosmetic emulsions with the addition of hydroalcoholic apple extract	The determination of each hydro-module, alcoholic extract obtained by sonication, reflux and several experimental parameters, as well as TPC, antioxidant capacity, SPF determination, and incorporation of the apple extract in a sunscreen emulsion and characterization, were carried out.	The optimum volume required to extract the maximum amount of soluble matter deriving from dried apples was 18 mL. By refluxing the highest amounts of polyphenols (5.02 mg gallic acid/g dry weight (DW), the highest antioxidant activity (75.76 mM Trolox/g DW), and the highest SPF (1.01), were obtained. After exposure to UV light for 120 min, SPF values decreased by up to 15%. The highest SPF was obtained for the emulsion that contained 40% apple extract.	2022	[61]
The purpose of this research was to formulate gels and emulgels containing a complex of phenolic compounds of apple extracts and to perform a biopharmaceutical evaluation of semi-solid pharmaceutical forms	Gels were formulated using different concentrations of carbomer. Authors chose to add 1% aqueous dry extracts of phenolic compounds to each prepared gel and emulgel formulation. Franz-type diffusion cells, DPPH and FRAP methods were exploited.	The in vitro release test revealed that the largest total amount of phenolics was released from formulation E5 and that the smallest amount was released from formulations G3 and G5. Avicularin was the predominant flavonoid. (–)-Epicatechin was the main phenolic compound. The largest amounts of individual phenolic compounds were released from the gel and emulgel formulations after 6 h. The antioxidant activity revealed that all the gel (G1–G6) and emulgel (E1–E6) formulations after 6 h were stronger compared to when these activities were observed in the formulations after 2 or 4 h.	2020	[62]
In vivo study				
The Annouka apple (AA) extract, rich in (UA) and sunflower oil (AAO) containing 784.40 ± 7.579 µg/mL of UA), was evaluated to inhibit porcine elastase enzymatic reactions via a validated spectrophotometric method	The analyzed standards and the extracts were 40 subjects aged 40 to 65 years with relaxed skin tone and dermis thinning who were eligible for the study. A mandatory washout period of 7 days was required before study entry. During the baseline (D_0_) and check-up visits, participants were assessed for the monitored skin parameters and randomly assigned to a treatment group. They had to apply about 2 mg of the assigned product on their face twice daily for 28 days. Before admission, they received a clinical skin examination and a detailed cosmetic questionnaire. Patients were randomly assigned AAO-based cream (2.5% *w*/*w* twice daily) or vehicle cream twice daily (placebo group) in a 1:1 ratio. Patients, and investigators remained masked to each patient’s treatment assignment throughout the study.	The formulated AAO was able to inhibit the elastase activity with a calculated IC_50_ of 286.42 mg/mL. The inhibitory activity of AAO towards elastase may be also potentiated by the activity of apolar polyphenols identified in AAO which are phloridzin (0.15 ± 0.010 mg/mL), phloretin (0.07 ± 0.01 mg/mL), rutin and quercetin-3-*O*-glucoside (0.76 ± 0.001 and 0.71 ± 0.007 mg/mL, respectively). Quercetin was generally proven to be the most active polyphenol. The formulated AAO-based product is safe and not an irritant for topical applications. In accordance with the in vitro results, the in vivo evidence indicates a relevant anti-aging effect in terms of the reduction in skin wrinkles at the nasolabial and T area levels in treated subjects. Additionally, a positive correlation between gross elasticity (R_2_) and hydration was calculated.	2024	[53]

**Table 4:** Apple pomace skin-protecting properties.

Hypothesis—Intervention	Study Design/Parameters Examined	Main Findings	Year of Study	Reference
In vitro and/or ex vitro studies				
The purpose of this study was to evaluate the sun protection factor (SPF) of cosmetic emulsions with the addition of hydroalcoholic apple extract	The determination of each hydromodule, alcoholic extract obtained by sonication, reflux and several experimental parameters, as well as TPC, antioxidant capacity, SPF determination, and the incorporation of the apple extract in a sunscreen emulsion and characterization, were carried out	The optimum volume required to extract the maximum amount of soluble matter deriving from dried apples was 18 mL. By refluxing the highest amounts of polyphenols (5.02 mg gallic acid/g dry weight (DW), the highest antioxidant activity (75.76 mM Trolox/g DW) and the highest SPF (1.01) were obtained. After exposure to UV light for 120 min, SPF values decreased by up to 15%. The highest SPF was obtained for the emulsion that contained 40% apple extract.	2022	[61]
The purpose of this research was to formulate gels and emulgels containing a complex of phenolic compounds of apple extracts and to perform a biopharmaceutical evaluation of semi-solid pharmaceutical forms	Gels were formulated using different concentrations of carbomer. Authors chose to add 1% aqueous dry extracts of phenolic compounds to each prepared gel and emulgel formulation. Franz-type diffusion cells, DPPH and FRAP methods were exploited.	The in vitro release test revealed that the largest total amount of phenolics was released from formulation E5 and that the smallest amount was released from formulations G3 and G5. Avicularin was the predominant flavonoid. (–)-Epicatechin, was the main phenolic compound. The largest amounts of individual phenolic compounds were released from the gel and emulgel formulations after 6 h. The antioxidant activity revealed that all the gel (G1–G6) and emulgel (E1–E6) formulations after 6 h were stronger compared to when these activities were observed in the formulations after 2 or 4 h.	2020	[62]
The aim of this study was to select a suitable extraction method and extraction agents which will provide the wild apple fruits extracts with the highest content of polyphenolic compounds and the most pronounced antioxidant activity	The determination of total flavonoid content (TFC) in the extracts was performed. TPC in the extracts was determined by colorimetric method with minor modifications Tannins were assessed (TT) using the vanillin assay. DPPH and FRAP tests were conducted.	Extracts with TPC ranging from 172.91 to 1556.99 mg GAE/100 g d.w., TFC from 3.97 to 182.22 mg RE/100 g d.w., TT from 72.73 to 8872.73 mg CE/100 g d.w. and TA from 0.70 to 14.63%. %RSC ranged from 8.12 to 78.43% RSC, FRAP value from 2.12 to 7.65 mM Fe^2+^ and % AOA from 57.73 to 95.32% AOA. Extracts made using 70% ethanol and distilled water and ultrasonic extraction showed good polyphenolic content and the best antioxidant activity.	2016	[68]
Some products and coproducts were analyzed, such as the cultivar ‘Fuji’, apple powder, concentrate apple juice and fruits from the pollination apple (*Malus everest*) plant	Samples were prepared in three replications and homogenized in an ultrawax homogenizer, and then centrifuged at 15,000 rpm and 4 °C temperature. After sample preparation, TPC and antioxidant activity were assayed as well. Different solvents and solvent mixtures, were employed in this assay.	Fruits from the pollination apple plant (*Malus everest*) showed the highest antioxidant activity among all the studied products and co-products. Comparing *Malus everest* and *Malus domestica*, the TPC was 8-fold and antioxidant activity was 38-fold superior in the *Malus everest.* Ethanol was the most efficient solvent, followed by acetone.	2012	[69]
The aim of this study was to determine antioxidant activity, total polyphenol and flavonoid content of apple juice enriched by water herbal extracts. Furthermore, the sensory traits of enriched apple juice were evaluated and addressed	Several variants of apple juice enriched by herbal extract were prepared: variant 1 (60% of apple juice + 40% of mint water extract), variant 2 (60% of apple juice + 40% of lemon balm water extract), variant 3 (60% of apple juice +40% of oregano water extract), variant 4 (60% of apple juice +40% of wild thyme water extract) and variant 5 (60% of apple juice + 40% of salvia water extract). DPPH, phosphomolybdenum method, TPC, TFC, sensory analysis, radical scavenging activity, reducing power, and statistical analysis, were carried out.	DPPH radical decreased in this order: apple juice with lemon balm > apple juice with salvia > apple juice with oregano water extract > apple juice with mint water extract > apple juice with wild thyme water extract > 100% apple juice. The power of the variants exhibited a reduction in the following order: apple juice with salvia > apple juice with lemon balm > apple juice with wild thyme > apple juice with oregano > apple juice with mint > 100% apple juice. TPC was highest in the following order: apple juice with oregano > apple juice with lemon balm > apple juice with salvia > apple juice with wild thyme > apple juice with mint > 100% apple juice. TFC was highest the following order: apple juice with salvia > apple juice with oregano > apple juice with lemon balm > apple juice with wild thyme > apple juice with mint > 100% apple juice.	2015	[70]
In vivo studies				
This study highlighted the mechanisms that flavonoids may promote survival or extend lifespan in some model organisms such as fruit flies, worms, and mice	Wild–type N2 and all transgenic strains, as well as CL2070 and CF1553, were utilized for measuring the subcellular localization of DAF-16. Resistance to environmental sensors, intracellular ROS levels, lifespan assay, fertility assay, age-related decline of muscle function measurement, stress-responsive gene expression, amyloid–beta-induced toxicity, high-glucose-diet-induced toxicity, the degeneration of dopaminergic neurons, gene knockdown by RNAi, and quantitative reverse transcription polymerase chain reaction (qRT-PCR) were evaluated. The feeding of RNAi clones from RNAi library to the wild-type laboratory strain N2 is a standard gene knockdown method. Three RNA clones, daf-16, skn-1 and bec-1, were used.	Dietary supplementation with phlorizin increased resistance to oxidative stress and UV radiation, while also extending lifespan and delaying the age-related decline of muscle function. Beneficial effects were displayed against diabetes mellitus, Alzheimer’s and Parkinson’s disease. Phlorizin enhanced the nuclear localization of DAF-16. Downstream targets of DAF-16, sod-3 and hsp-16.2 exhibited increased expression by phlorizin in vivo. qRT-PCR analysis showed an upregulation of ctl-1 and sod-3, antioxidant genes that are regulated by DAF-16. The lifespan extension conferred by phlorizin completely disappeared in age-1 mutant, in which lifespan increased via reduced insulin/IGF-1-like signaling and DAF-16 is required for the insurance lifespan extension. The phlorizin-induced lifespan extension overlaps with the long lifespan by reduced insulin/IGF-1-like signaling.	2022	[71]
In this study, various assays, i.e., the DPPH radical scavenging assay and the ABTS assay, were performed to evaluate the antioxidant activity of the extracts of leaf and branch of *S. samarangense*, and the safety of the extracts was shown via the MTT assay and human skin primary irritation test	This test was performed on 34 men or women between 20 and 60 years of age who met the criteria for subject selection. The *S. samarangense* used in this study were separated from leaves and branches that were extracted at 65 °C for 8 h in distilled water. DPPH, ABTS and dimethyl-4–phenylene –diamine (DMPD) radical scavenging activity, as well as nitrite scavenging activity, ferrous-ion chelating activity, cupric reducing antioxidant capacity (CUPRAC), reducing power assay, FRAP assay, TPC, TFC, cell cultures, cell viability, skin primary irritation test, and HPLC fingerprint, were conducted and assessed. The primary skin irritation response was evaluated. The skin reaction results for each test substance were calculated from an appropriate formula. The average reactivity of each calculated test substance was also determined.	The IC_50_ value of DPPH was 4 times larger for the leaf and branch extracts than ascorbic acid, while the value concerning ABTS was similar for these three agents, and was 13 times larger for DMPD for the leaf and branch extracts than ascorbic acid. The IC_50_ value of ferrous-ion chelating activity was concurrently 10 times larger for the leaf and branch extracts than eethylenediaminetetraacetic acid (EDTA). This value of the nitrite scavenging activity, was 20 times larger than that of ascorbic acid, and with the FRAP, CUPRAC, and reducing power assay, it was 10 times larger for the leaf and branch extracts than that of ascorbic acid. TPC was ~1.1 greater in the branch rather than the leaf extract and the TFC was ~1.9 greater. The cell viability of leaf and branch extracts, using human keratinocyte (HaCaT), was slightly lowered from 92 to 64% in the leaf and from 92 to 77% in the branch, indicating lower toxicity than the leaf. Slight irritation was observed in the human skin primary irritation test.	2020	[72]

**Table 5:** Apple pomace antioxidant activity.

Hypothesis—Intervention	Study Design/Parameters Examined	Main Findings	Year of Study	References
In vitro and/or ex vitro studies				
The purpose of this research was to formulate gels and emulgels containing a complex of phenolic compounds of apple extracts and to perform a biopharmaceutical evaluation of semi-solid pharmaceutical forms	Gels were formulated using different concentrations of carbomer. Authors chose to add 1% aqueous dry extracts of phenolic compounds to each prepared gel and emulgel formulation. Franz-type diffusion cells, DPPH and FRAP methods were exploited.	The in vitro release test revealed that the largest total amount of phenolics was released from formulation E5 and that the smallest amount was released from formulations G3 and G5. Avicularin was the predominant flavonoid. (–)-Epicatechin was the main phenolic compound. The largest amounts of individual phenolic compounds were released from the gel and emulgel formulations after 6 h. The antioxidant activity revealed that all the gel (G1–G6) and emulgel (E1–E6) formulations were stronger after 6 h compared to when these activities were observed in the formulations after 2 or 4 h	2020	[62]
This study used an experimental method from gel mask formulation with juice and the ultrasonic extraction of green apple fruit	Formula I was a peel-off mask with juice extract and formula II was a peel-off mask with ultrasonic extract. A total of 2 g of green apple juice extract and 2 g of green apple maceration extract was added to each peel-off gel base mask. Ultrasonic extraction was performed via ethanol to extract the juice. The peel-off mask was tested for antioxidant activity by UV–Vis spectrophotometry with the DPPH method. pH test and antioxidant activity tests were conducted as well.	PVA aided in providing a peel-off effect due to its adhesive properties, enabling film-layer formation. CMC is a gelling agent and may increase fluid resistance from the viscosity of the solution, so as to form a mass compact gel. The pH of formula I was 6, and for formula II it was 4.93, so the peel-off formula met the pH requirements and did not cause irritation when applied to the skin (pH range on topical skin formulations (4.5–6.5)). Formula I was 30.544 ppm; and formula II was 9.771 ppm—formula II had stronger antioxidant activity that formula I. According to the IC_50_ value parameter, the quercetin and peel-off mask formula is a very strong antioxidant (IC_50_ value < 50).	2022	[79]
This study investigated the use of phenolic compounds extracted from the new Korean apple cultivar Arisoo in cosmetics and food additives	Two different extraction methods were used (water and ethanol). The water extract was prepared with 1 g of fruit powder, mixed with 200 mL of distilled water, boiled until it reached a total volume of 100 mL, then cooled and stirred for 24 h. The ethanol extract was prepared with 1 g of fruit powder, mixed with 100 mL of 10–100% ethanol, and stored at 4 °C for 24 h, with shaking. The measurement of inhibitory activities of apple extracts against elastase and collagenase, as well as α-amylase and α-glucosidase, along with statistical analysis, were conducted. Both extracts were filtered through filter paper, concentrated and stored at 4 °C prior to use or analysis. Total phenolic contents of water and ethanol extracts were measured by using the method described by Folin and Denis.	Water- and ethanol-extracted phenolic compound yields were 1.84 and 2.14 mg/g fresh weight, respectively. In the antioxidant test, 100 μg/mL of water- and ethanol-extracted phenolic compounds increased the scavenging activity of the DPPH radical by 91.86% and 87.44%, and showed antioxidant protection factors of 2.80 and 1.79. Phenolic extracts derived from Arisoo apples have better anti–wrinkle activity than Fuji apples. The inhibitory activities of WEP and EEP on yeast α-glucosidase were tested to assess their anti-diabetic activities. WEP and EEP from Arisoo apples inhibited 83.92% and 100.0% of the α-glucosidase activity, whereas those from Fuji apples inhibited 63.33% and 92.84%, respectively, suggesting that Arisoo extracts have better anti-diabetic activity.	2019	[54]
The aim of this study is to prepare a stable water and oil emulsion (*w*/*o*) from the extract (3%) of *Malus domestica* containing high percentage of antioxidant activity	In this study, different solvents were used for the extraction of flavonoids from fruit, as follows: (1) 80% (*v*/*v*) acetone in double-distilled water, (2) 70% (*v*/*v*) ethanol in double-distilled water 0.01% (*v*/*v*) HCl, (3) 70% (*v*/*v*) methanol in double-distilled water 0.01% (*v*/*v*) HCl, methanol: formic acid: water (MFW: 70:2:28) with high antioxidant percentage. The determination of the antioxidant activity (DPPH), properties and types of emulsion evaluation, physical analysis (color, thickness, look, feel) tests, microscopic tests, pH determination, electrical conductivity measurement, centrifugation tests, and stability tests, were conducted, altogether. Formulation and preparation techniques, and in vitro characterization methods of emulsions, altogether, were carried out. Stability studies of this emulsion were carried out, and different storage conditions were applied.	A slight liquefaction was observed in formulation samples kept at 40 °C and 40 °C + 75% RH at 28th day of observation period. No phase separation was displayed in samples kept at 8 °C, 25 °C, 40 °C and 40 °C + 75% RH, over a period of 28 days. The base and the formulation were stable at all storage conditions for 28 days. No electrical conductivity was displayed in samples kept at 8 °C, 25 °C, 40 °C and 40 °C + 75% RH, in the period of 28 days, which may be attributed to the non-conductive nature of the oil phase. The pH values of samples kept at 8 °C, 25 °C, 40 °C and 40 °C + 75% RH, over a period of 28 days, decreased, possibly due to the by-products generated by the degradation.	2010	[76]
This study aims at assessing the antioxidant capacity of apple peel flavonoids in vegetable juices as the food model and evaluating the contribution of the hydrophilic and lipophilic antioxidants to the total antioxidant capacity equivalent to ascorbic acid using DPPH and FRAP assays	The extraction of peel flavonoids was carried out using 80% aqueous ethanol (*v*/*v*), while photo diode array (PDA)-HPLC-RP analysis, the characterization of proanthocyanins, the enrichment of vegetable juice with bottled tomato and carrot juices, antioxidant capacity, lipid peroxidation analysis, FRAP assay and DPPH assay were conducted and statistically analyzed. The antioxidant contribution of the added extract was measured as ascorbic acid equivalents with ferric-reducing antioxidant power and radical scavenging capacity against DPPH assays, and as inhibition against lipid peroxidation using an emulsified lipid-in-an-oven test.	The hydrophilic components of the enriched juices with peel flavonoids, contributed highly to the antioxidant activity. Added peel phenolic hydrophilic components, were significantly higher than lipophilic counterparts. The addition of apple peel flavonoids at concentrations equal to or above 160 mg gallic acid equivalents (GAEs)/L in terms of total phenolics in the juices led to significantly higher (*p* < 0.05) radical scavenging capacity and to an increased protection against lipid peroxidation compared to control. The oxidative index of the model emulsified lipid with added enriched juices (20 mg/L of GAEs) was lower than the control	2016	[80]
In vivo studies				
This study highlighted the mechanisms that flavonoids may promote survival or extend lifespan in some model organisms such as fruit flies, worms, and mice	Wild–type N2 and all transgenic strains, as well as CL2070 and CF1553, were utilized for measuring subcellular localization of DAF-16. Resistance to environmental sensors, intracellular ROS levels, lifespan assay, fertility assay, age-related decline of muscle function measurement, stress-responsive genes expression, amyloid–beta-induced toxicity, high-glucose-diet-induced toxicity, degeneration of dopaminergic neurons, gene knockdown by RNAi, and quantitative reverse transcription polymerase chain reaction (qRT-PCR) were evaluated. The feeding of RNAi clones from RNAi library to the wild-type laboratory strain N2 is a standard gene knockdown method Three RNA clones, daf-16, skn-1 and bec-1, were used.	Dietary supplementation with phlorizin increased resistance to oxidative stress and UV radiation, while also extending lifespan and delaying the age-related decline of muscle function. Beneficial effects were displayed against diabetes mellitus, Alzheimer’s and Parkinson’s disease. Phlorizin enhanced the nuclear localization of DAF-16. Downstream targets of DAF-16, sod-3 and hsp-16.2 exhibited increased expression by phlorizin in vivo. qRT-PCR analysis showed an upregulation of ctl-1 and sod-3, antioxidant genes that are regulated by DAF-16. The lifespan extension conferred by phlorizin completely disappeared in age-1 mutant, in which the lifespan increase via reduced insulin/IGF-1-like signaling and DAF-16 is required for the insurance lifespan extension. The phlorizin-induced lifespan extension overlaps with the long lifespan induced by reduced insulin/IGF-1-like signaling.	2022	[71]
In this study, various assays,, i.e., DPPH radical scavenging assay and the ABTS assay, were performed to evaluate the antioxidant activity of the extracts of leaf and branch of *S. samarangense*, and the safety of the extracts was shown via the MTT assay and human skin primary irritation test	This test was performed on 34 men or women between 20 and 60 years of age who met the criteria for subject selection. The *S. samarangense* used in this study were separated from leaves and branches that were extracted at 65 °C for 8 h in distilled water. DPPH, ABTS and dimethyl-4–phenylene –diamine (DMPD) radical scavenging activity, as well as nitrite scavenging activity, ferrous-ion chelating activity, cupric reducing antioxidant capacity (CUPRAC), reducing power assay, FRAP assay, TPC, TFC, cell cultures, cell viability, skin primary irritation test, and HPLC fingerprint, were conducted and assessed. The primary skin irritation response was evaluated. The skin reaction results for each test substance were calculated from an appropriate formula. The average reactivity of each calculated test substance was also determined.	The IC_50_ value of DPPH was 4 times larger for the leaf and branch extracts than ascorbic acid, while this value concerning ABTS was similar for these three agents, and was 13 times larger for DMPD for the leaf and branch extracts than ascorbic acid. The IC_50_ value of ferrous-ion chelating activity was concurrently 10 times larger for the leaf and branch extracts than ethylenediaminetetraacetic acid (EDTA). This value of the nitrite scavenging activity was 20 times larger than that of ascorbic acid, and for the FRAP, CUPRAC, and reducing power assay, it was 10 times larger for the leaf and branch extracts than that of ascorbic acid. TPC was ~1.1 greater in the branch rather than the leaf extract, and for TFC, ~1.9 greater. The cell viability of leaf and branch extracts, using human keratinocyte (HaCaT), was slightly lowered from 92 to 64% in the leaf and from 92 to 77% in the branch, indicating lower toxicity than the leaf. Slight irritation was observed in the human skin primary irritation test.	2020	[72]
The effects of apple polyphenols on hyperlipidemia, atherosclerosis, hepatic steatosis and endothelial function and investigated the potential mechanisms, were evaluated	A total of 30 12-week-old male ApoE^−/−^ mice with a C57BL/6J background were used and fed with a Western-type diet (21% fat from plant sources, 0.2% cholesterol) when they were 12 weeks old. The mice were randomly divided into three groups: a vehicle control group (WD, equivalent vehicle), an atorvastatin group (ATO, 10 mg/kg/day) and an apple polyphenol (AP) group (APs, 100 mg/kg/day), in which the designated treatment was administered by the intragastric route. After 12 weeks of treatment, the mice were sacrificed, and the tissues were collected and immediately frozen. Quantification of the percentage of aortic atheroma was performed. The macrophage content of the atherosclerotic plaque was visualized Immunohistochemistry, assessments of aortic atherosclerosis and hepatic histology, hepatic redox status, measurements of body weight, hepatic lipids, and metabolic parameters, cell isolation and adhesion assay, cell viability assay and ROS detection, quantitation of gene expression, enzyme-linked immunosorbent assay, as well as Western blotting, were carried out. The liver tissues were also used for the measurement of oxidative activity and quantitative RT-PCR, for the associated hepatic lipogenic genes.	The APs significantly increased the plasma levels of high-density lipoprotein (HDL) cholesterol and markedly upregulated the glutathione peroxidase (GPx), catalase (CAT) and superoxide dismutase (SOD) levels in liver tissues. The AP treatment modulated lipid metabolism. Histological assessment showed that the APs treatment also reduced macrophage infiltration in the aortic root plaque and the inflammatory cells infiltrations into the liver tissues. The AP treatment greatly reduced the ox-LDL-induced endothelial dysfunction and monocyte adhesion to rat aortic endothelial cells (RAECs). The AP treatment suppressed the ROS/MAPK/NF-κB signaling pathway, and consequently, reduced CCL-2, ICAM-1 and VCAM-1 expression. The APs are a beneficial nutritional supplement for the attenuation of atherosclerosis.	2015	[81]

## Abbreviations

Adenosine 5′-DipPhosphate (ADP); Alpha Linolenic Acid (ALA); 2,2′-Azinobis-(3-Ethylbenzthiazolin-6-Sulfonic Acid) assay (ABTS assay); Cycloxigenase (COX); Cytoplasmic PhosphoLipase A2 (cPLA); Fatty Acid (FA); G-protein Coupled Membrane Receptors (GPCMR); Inhibitor of NF-kB (IKB); Inositol TrisPhosphate (IP3); Linoleic Acid (LA); Mitogen-activated protein kinase (MAPK); Monounsaturated Fatty Acid (MUFA); Nuclear Factor kappa beta (NF-kB); Oleic Acid (OA); PAF-cholinephsphotransferase and lyso-phosphatidylcholine acetyltransferase 2 (LPCAT); PAF-receptor (PAF-R); PhosphatidylCholines (PC); PhosphatidylEthanolamines (PE); Platelet Activating Factor (PAF); Polyunsaturated Fatty Acid (PUFA); Protease-Activated Receptors for Thromin (PAF-CPT); Reactive Oxygen Species (ROS); Saturated Fatty Acid (SFA); Unsaturated Fatty Acid (UFA).
